# Ischiofemoral impingement in joint preserving hip surgery: prevalence and imaging predictors

**DOI:** 10.1186/s13244-025-01946-2

**Published:** 2025-04-04

**Authors:** Alexander F. Heimann, Moritz Wagner, Peter Vavron, Alexander Brunner, Till D. Lerch, Ehrenfried Schmaranzer, Joseph M. Schwab, Simon D. Steppacher, Moritz Tannast, Reto Sutter, Florian Schmaranzer

**Affiliations:** 1 Department of Orthopaedic Surgery, HFR—Cantonal Hospital, University of Fribourg, Fribourg, Switzerland; 2 Center for Computer Assisted & Reconstructive Surgery, New England Baptist Hospital, Boston, MA USA; 3 Department of Orthopaedic Surgery, District Hospital, St. Johann in Tirol, Austria; 4 Department of Diagnostic-, Interventional- and Pediatric Radiology, Inselspital, Bern University Hospital, University of Bern, Bern, Switzerland; 5 Department of Radiology, District Hospital, St. Johann in Tirol, Austria; 6 Department of Orthopaedic Surgery, Inselspital Bern, University Hospital, University of Bern, Bern, Switzerland; 7 Department of Radiology, Balgrist University Hospital, Faculty of Medicine, University of Zürich, Zurich, Switzerland

**Keywords:** Hip, MRI, Femoroacetabular impingement, Ischiofemoral impingement, Hip arthroscopy

## Abstract

**Objectives:**

To determine the prevalence of ischiofemoral impingement (IFI) in young patients evaluated for joint-preserving hip surgery and investigate its associations with osseous deformities and intra-articular pathologies.

**Methods:**

Retrospective study of 256 hips (224 patients, mean age 34 years) that were examined with radiographs and MR arthrography for hip pain. Quadratus femoris muscle edema was used to indicate IFI and measurements of ischiofemoral space were performed. Imaging analysis assessed cam deformity, femoral torsion, neck-shaft angle, ischial angle, acetabular coverage-/ version, and chondro-labral pathology. Prevalence of MRI findings consistent with IFI was calculated and univariate- and multivariate logistic regression identified associations between IFI and hip deformities.

**Results:**

Quadratus femoris muscle edema consistent with IFI was present in 9% (23/256 hips) with narrowing of the ischiofemoral distance (1.7 ± 0.6 cm vs 2.8 ± 0.7 cm in the control group, *p* < 0.001) and a higher prevalence in females (89% vs 45%, *p* < 0.001). Multiple regression identified female sex (OR 12.5, 95% CI: 1.6–98.2, *p* = 0.017), high femoral torsion (OR 3.9, 1.4–10.4, *p* = 0.008), and ischial angle > 127° (OR 5.9, 1.3–27.1, *p* = 0.023) as independent predictors of IFI. Labral tears were highly prevalent in both IFI and control groups (87% vs 89%, *p* = 0.732); cartilage lesions were less common in the IFI group (26% vs 52%, *p* = 0.027).

**Conclusion:**

IFI was present in 9% of young patients evaluated for joint-preserving surgery, associated with female sex, high femoral torsion and increased ischial angle. The comparable prevalence of labral lesions but lower prevalence of cartilage damage suggests complex relationships between extra- and intra-articular pathologies.

**Critical relevance statement:**

Recognizing IFI and its link to hip deformities and chondrolabral damage is crucial for clinicians, as it represents an important differential diagnosis, directly impacting joint-preserving treatment strategies in young adults with hip pain.

**Key Points:**

- The prevalence and imaging predictors of IFI in young patients remain unknown.
- IFI occurred in 9%, with predictors including female sex, high femoral torsion, and an increased ischial angle.
- IFI is an important differential diagnosis in joint-preserving hip surgery.

**Graphical Abstract:**

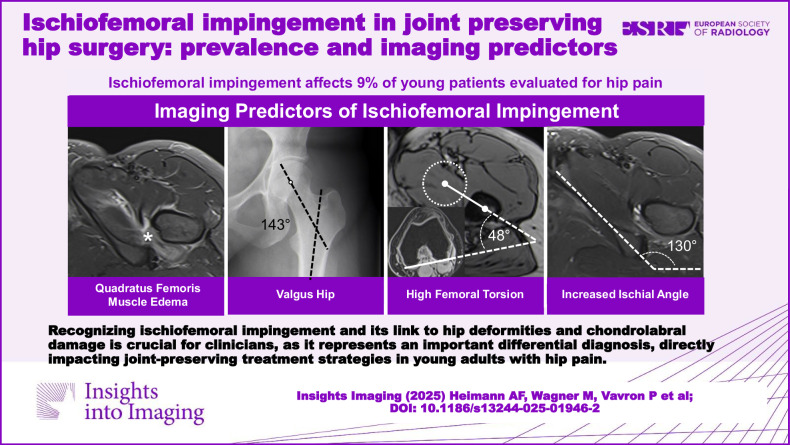

## Introduction

Ischiofemoral impingement (IFI) is an extra-articular compression phenomenon characterized by the narrowing of the space between the ischium and the proximal femur. First described in 2008 [1], IFI has gained increasing recognition as a cause of hip pain, particularly in adult females [2–5].

IFI is defined by a combination of clinical symptoms, including deep gluteal pain and occasional sciatica, along with imaging findings of ischiofemoral space narrowing and quadratus femoris muscle abnormalities [6].

While our understanding of IFI has grown over the past 15 years, several aspects of this condition remain unclear, particularly in younger patient populations. The prevalence of IFI findings in young patients being evaluated for joint-preserving hip surgery is not well understood. Given that older patients are generally not considered for joint-preserving procedures, our study focuses on the prevalence of IFI in this specific demographic. Furthermore, the potential associations between IFI and other hip pathologies, such as osseous deformities and chondrolabral damage, have not been thoroughly investigated in these patients. Recent studies have highlighted the multifactorial etiology of IFI, involving factors such as lumbar spine alignment, hip abductor muscle status, pelvic configuration, and hip and femoral morphology [7].

These findings suggest that IFI may coexist with or potentially contribute to other hip pathologies. However, the relationship between IFI and conditions commonly addressed in joint-preserving hip surgery, such as femoroacetabular impingement (FAI) or hip dysplasia, remains poorly understood. The aim of this study is twofold: (1) to assess the prevalence of MRI findings consistent with IFI in young patients being evaluated for joint-preserving hip surgery, and (2) to investigate the potential associations between these IFI findings and the presence of osseous hip deformities and chondrolabral damage. By elucidating these relationships, we hope to enhance our understanding of IFI in the context of other hip pathologies and potentially inform more comprehensive treatment strategies for young patients with hip pain.

## Materials and methods

### Study design and participant inclusion

**Fig. 1:**
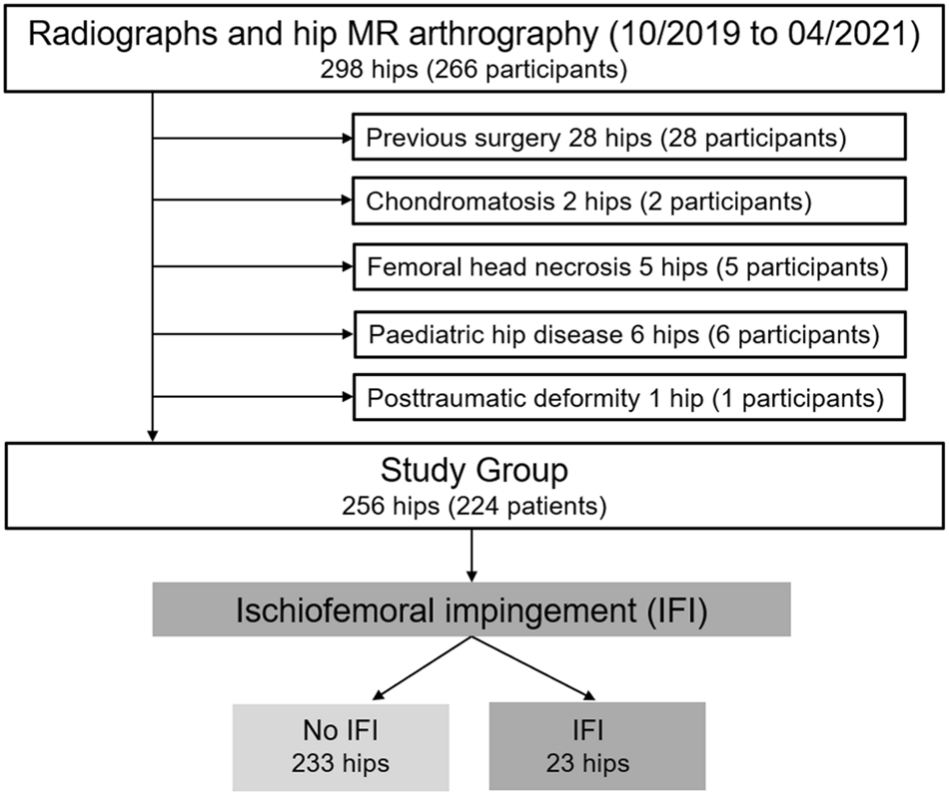
Study flowchart of young patients (mean age 34 ± 11 years) eligible for joint preserving surgery who were stratified in groups with and without imaging findings of IFI

This study was a retrospective, observational analysis, approved by the institutional review board at a primary referral center for joint-preserving hip surgery in Austria, and conducted with a waiver of written informed consent. The cohort included consecutive patients who sought care at our outpatient orthopedic clinic for hip and/or buttock pain between October 2019 and April 2021. All patients underwent imaging of the symptomatic hip. Exclusion criteria were prior hip surgery, femoral head necrosis, pediatric hip disease (Legg Calve Perthes disease, slipped capital femoral epiphysis), posttraumatic deformities, and synovial chondromatosis. A total of 256 hips from 224 participants were assessed, with the cohort divided based on the presence or absence of quadratus femoris muscle edema as an indicator of IFI (Fig. 1).

### Radiographic imaging and hip MR arthrography

Standardized imaging protocols were employed for all participants. Each patient underwent anteroposterior (AP) pelvic radiographs and 45° modified Dunn views of the affected hip.

Preoperative magnetic resonance arthrography (MRA) using a large, flexible body coil was performed at 1.5 T (Aera; Siemens Healthcare), with intra-articular injection of 15–20 mL of 0.9% NaCl solution [8]. Injection was performed under fluoroscopic guidance using 1–2 mL iodinated contrast agent (iopamidol, 200 mg/mL; Iopamiro 200; Bracco) and an additional injection of 2–5 mL local anesthetic (ropivacaine hydrochloride; 2 mg/mL; Ropinaest; Gebro Pharma). Leg traction during MRI was applied according to our institutional protocol utilizing a previously described approach and traction device (TRACView; Menges Medical) [9]. The imaging protocol included axial short-tau inversion recovery (STIR) sequences and 3D T1-weighted volumetric interpolated breath-hold examination DIXON sequences of the pelvis and distal femoral condyles acquired without traction. Multiplanar proton-density weighted turbo spin echo images were acquired under leg traction in coronal, axial-oblique, sagittal, and radial planes to identify intra-articular lesions. (Supplementary Table 1).

### Image analysis

**Fig. 2:**
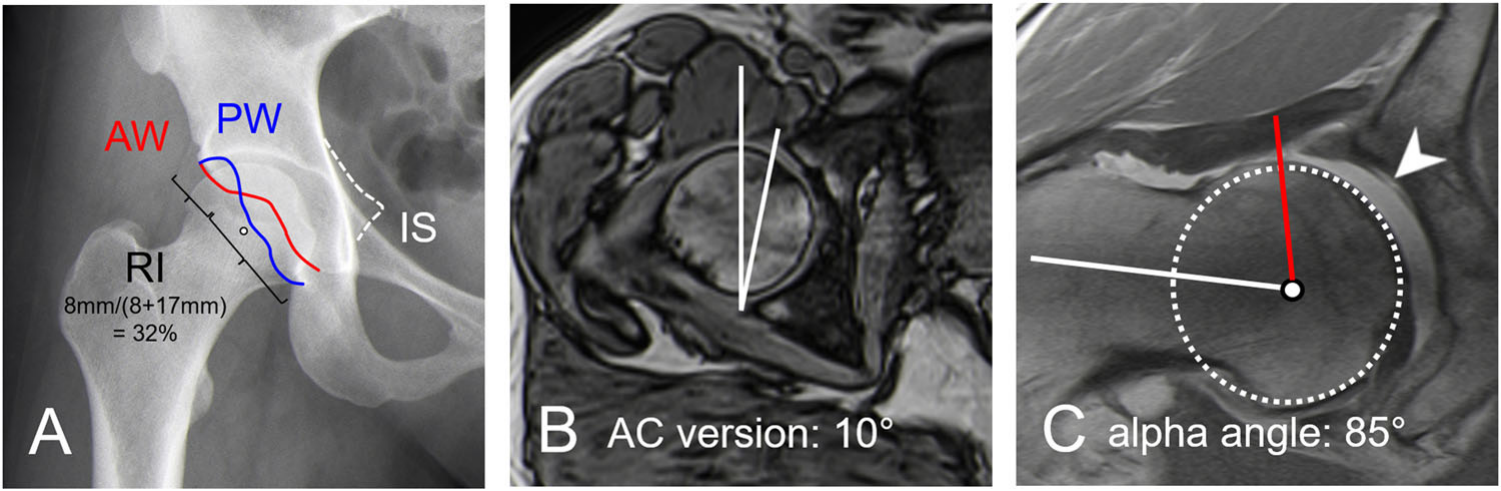
**A** AP pelvic radiograph shows acetabular retroversion with a positive cross-over sign with an anterior wall (AW) projecting lateral to the posterior wall (PW), the extension of the retroverted part relative to the entire acetabulum is reflected by the retroversion index (RI of 32%); positive posterior wall sign with a projection of the posterior wall (PW) medial to the femoral head center and positive ischial spine sign with the projection of the ischial spine (IS) into the pelvic inlet. **B** Measurement of the acetabular version of 10° at the level of the center of the femoral head is shown on axial T1-w volume interpolated breath-hold examination DIXON sequence. **C** Measurement of alpha angle of 85° on a radial proton-density weighted turbo spin echo image indicating the presence of a cam deformity, note delamination of the acetabular cartilage (arrowhead)

**Fig. 3:**
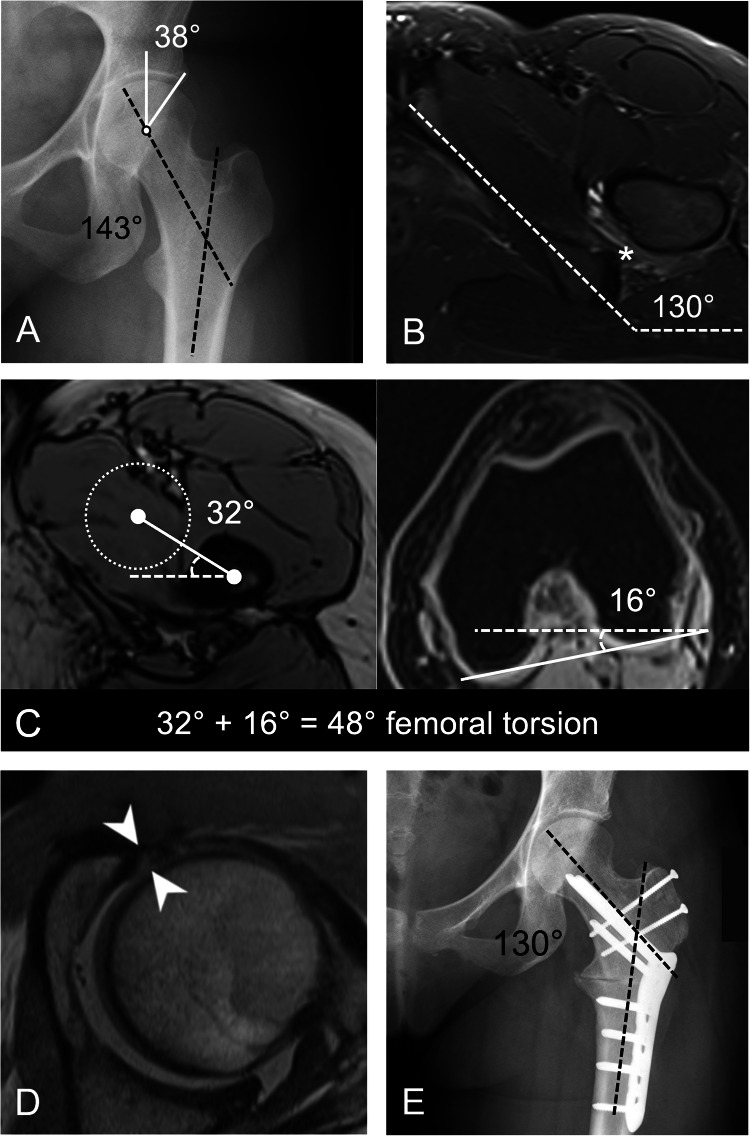
AP pelvic radiograph of a 25-year-old female patient with left buttock pain and positive posterior impingement test. Diagnostic imaging revealed (**A**) a valgus deformity (neck shaft angle of 143°) and acetabular overcoverage (LCE 38°). The patient underwent subsequent hip MRI which showed (**B**) increased ischial angle and edema in the quadratus femoris muscle (asterisk) on axial STIR MR-image, (**C**) increased femoral torsion (48°) measured on axial T1-w volume-interpolated breath-hold examination DIXON MR-images. **D** The axial oblique proton-density weighted turbo spin echo MR arthrogram showed an intrasubstance tear of the anterosuperior labrum (arrowheads) without concomitant cartilage damage. Overall this led to the clinical diagnosis of IFI. The patient underwent subsequent (**E**) surgical hip dislocation with additional intertrochanteric varisation-derotational osteotomy with good relief of symptoms 6 months after surgery

Image analysis was conducted by an experienced radiologist (E.S.) with 12 years of experience in hip imaging, following the Lisbon agreement guidelines for FAI assessment [10, 11]. Quadratus femoris muscle edema, identified by increased signal intensity on axial STIR images [6], was used to categorize the study participants into groups with and without imaging findings of IFI. Ischiofemoral distance was measured as the shortest distance between the lateral cortex of the ischial tuberosity and the medial cortex of the lesser trochanter [6]. On an AP pelvis view measured radiographic parameters included acetabular coverage (lateral center edge angle [LCE]), acetabular retroversion signs (cross-over, posterior wall, and ischial spine sign, and femoral neck-shaft angle (Figs. 2 and 3). MRI measurements of femoral torsion were performed on axial 3D T1-weighted volume interpolated breath-hold examination Dixon sequences per the method by Murphy et al [12]. The acetabular version was measured at the midlevel of the femoral head [13], while the ischial angle was used to assess the orientation of the inferior pubic ramus [2]. Alpha angles were measured on radial MRI [14] (Fig. 2).

The presence and extent of intraarticular degeneration were assessed and compared between groups, including presence/absence of labral tears, type of labral tear (base tear, intrasubstance/complex tear), presence/absence of cartilage damage, and presence/absence of a ligamentum teres lesion [10, 11]. Cartilage damage was routinely assessed using the following parameters: location, surface side (acetabular or femoral), and extent (< 2 h/> 2 h on the acetabular clockface) [10, 11]. Extensive cartilage damage was defined by extent (> 2 h on the clockface), rather than depth/type, as this has been associated with worse outcomes following arthroscopic FAI surgery. Similarly, the presence of extensive labral damage was assessed given its previously reported prognostic relevance [15, 16].

### Association female sex, osseous deformities, and IFI

The association between IFI and the following pelvic deformities was assessed: Hip dysplasia (LCE < 25°) [10], acetabular overcoverage (LCE > 35°), acetabular retroversion (retroversion index > 30%, posterior wall sign, and ischial spine sign positive) [17], and increased ischial angle (> 127°, refer to statistical analysis).

The association between IFI and the presence of a cam deformity (alpha angle > 60°) [14], valgus deformity (neck shaft angle > 135°) [10], and excessively high femoral torsion (> 35°) [13] was evaluated. In addition, female sex was included as it is a known risk factor for IFI in middle-aged women [2].

### Statistical analysis

Statistical analysis was performed using MedCalc (MedCalc Statistical Software, version 20.106, MedCalc Software Ltd, Ostend, Belgium). Normal distribution testing using a Kolmogorov-Smirnov test was carried out.

Depending on normal distribution testing, a comparison of the radiologic parameters between hips with/without IFI was performed using an independent samples *t*-test/Mann–Whitney test. A comparison of dichotomous parameters in hips with/without IFI was performed using chi-square tests. To investigate the relationship between IFI, sex, and osseous deformities, single-factor logistic regression analysis with calculation of the odds ratio (OR) and 95% confidence intervals (CI) was performed. Due to the lack of established thresholds for the ischial angle which is needed to assess its association with IFI, a receiver operating characteristic curve (ROC) was calculated: This yielded a cut-off value of 127° with a sensitivity of 91% and a specificity of 56%.

Subsequently, stepwise multiple logistic regression analysis was performed for the retained factors. To determine the interobserver reliability, a fellowship-trained musculoskeletal radiologist with seven years of experience in hip imaging (F.S.) evaluated the presence/absence of chondrolabral damage and ligamentum teres lesions on MRA in 100 randomly chosen patients (100 hips). Using Cohen’s kappa (*κ*), interpretation of interobserver agreement was performed as follows: *ĸ* values ≤ 0 as indicating no agreement, 0.01–0.20 none to slight, 0.21–0.40 fair, 0.41–0.60 moderate, 0.61–0.80 substantial, and 0.81–1.00 almost perfect agreement [18].

## Results

### Patient characteristics and prevalence of IFI

Of the 298 hips (266 participants), we excluded 42 hips (42 participants) due to: previous surgery (28 hips), synovial chondromatosis (2 hips), avascular necrosis of the femoral head (5 hips), pediatric hip disorders (4 hips with Legg Calve Perthes disease and 2 hips with slipped capital femoral epiphysis hips), or posttraumatic deformity (1 hip).

This yielded a total of 256 hips, with 23 hips in the IFI group and 233 hips in the control group yielding a prevalence of MRI findings of IFI of 9% (Fig. 1). The IFI group (1.7 ± 0.6 cm, 95% CI: 1.4–1.9 cm) demonstrated narrowing (*p* < 0.001) of the ischiofemoral space compared to the control group (2.8 ± 0.7 cm, 95% CI: 2.7 –2.9 cm). The mean age of the entire cohort was 34 ± 11 years, with no significant difference between groups (*p* = 0.759). There was a significant difference in sex distribution between the groups (*p* < 0.001), with females comprising 89% (95% CI: 67–99%) of the IFI group compared to 45% (38–52%) of the control group (Table 1). Joint preserving hip surgery was performed in 83 hips (32%). Hip arthroscopy was performed in 73 hips (29%), and surgical hip dislocation was performed in 3 hips (1%). Proximal derotational osteotomy of the femur was performed in 3 hips (1%) as an isolated procedure, or concomitant to hip arthroscopy or surgical hip dislocation in 2 hips (1%), respectively.

**Table 1:** Demographic characteristics of the study groups

Parameter	Overall (256 hips)	95% CI	IFI group (23 hips)	95% CI	Control group (233 hips)	95% CI	*p* value
Age (y), mean ± SD	34 ± 11	33–36	34 ± 12	29–39	34 ± 10	33–36	0.759
Female sex	109 (49)	42–55	17 (89)	67–99	92 (45)	38–52	**<** **0.001**
Bilateral	32 (14)	10–20	4 (21)	6–46	28 (14)	9–19	0.505

Values are depicted as *n* (%) if not otherwise noted

*IFI* ischiofemoral impingement, *SD* standard deviation

Bold highlights *p* values reaching statistical significance (*p* < 0.05)

### Association between osseous deformities and IFI

We found significant differences in hip morphology between the IFI and the control group (Table 2). The IFI group demonstrated a notably higher ischial angle of 132 ± 4° (95% CI: 130°–133°) compared to the control group’s 126 ± 9° (95% CI: 125°–128°), *p* < 0.001 (Fig. 3). This was further supported by the logistic regression analysis, which showed that an ischial angle > 127° was a significant independent predictor of IFI (OR 5.9, 95% CI: 1.3–27.1, *p* = 0.023 in multiple regression analysis, Table 3 and Fig. 3). High femoral torsion was more prevalent in the IFI group at 70% (95% CI: 47–87%) compared to 24% (95% CI: 19–31%) in the control group, *p* < 0.001, and was as well an independent factor in the regression analysis (OR 3.9, 95% CI: 1.4–10.4, *p* = 0.008 in multiple regression analysis, Table 3 and Fig. 3). The IFI group also exhibited a higher prevalence of valgus hip at 30% (95% CI: 13–53%) vs 11% (95% CI: 7–15%) in the control group, *p* = 0.014, which was significant in single factor analysis (OR 3.6, 95% CI: 1.4 to 9.7, *p* = 0.009) but not in multiple regression (Table 3 and Fig. 3). Cam deformity was less prevalent in the IFI group at 35% (95% CI: 16–57%) compared to 63% (95% CI: 56–69%) in the control group (*p* = 0.013). A negative association was also observed in the single factor regression analysis (OR 0.3, 95% CI: 0.1–0.8, *p* = 0.012), although it was not significant in the multiple regression model. Notably, the female sex emerged as an independent predictor of IFI in the multiple regression analysis (OR 12.5, 95% CI: 1.6–98.2, *p* = 0.017, Table 3).

**Table 2:** Comparison of osseous deformities between hips with vs without MRI findings of IFI

Parameter	IFI group (23 hips)		Control group (233 hips)		*p* value
	Mean ± SD	95% CI	Mean ± SD	95% CI	
Hip dysplasia (< 25°), n (%)	5 (22)	7–44	63 (27)	21–33	0.805
Acetabular overcoverage (> 35°), *n* (%)	4 (17)	5–39	45 (19)	14–25	1.00
LCE, °	29 ± 7	26–32	29 ± 8	28–30	0.971
Acetabular index, °	7 ± 6	4–9	7 ± 5	7–8	0.636
Acetabular version, °	21 ± 7	18–24	18 ± 6	17–18	**0.028**
Ischial angle, °	132 ± 4	130–133	126 ± 9	125–128	**<** **0.001**
Acetabular retroversion, *n* (%)^*^	1 (4)	0–22	15 (6)	4–10	1.00
Cross-over sign	10 (43)	23–66	67 (29)	23–35	0.152
Posterior wall sign	11 (48)	27–69	106 (45)	39–52	0.830
Ischial spine sign	8 (35)	16–57	58 (25)	19–31	0.321
Cam deformity (> 60°), *n* (%)	8 (35)	16–57	146 (63)	56–69	**0.013**
Alpha angle, °	60 ± 8	56–63	67 ± 11	66–69	**0.001**
Valgus hip (> 135°), *n* (%)	7 (30)	13–53	25 (11)	7–15	**0.014**
Neck shaft angle, °	133 ± 5	131–135	129 ± 8	128–130	**0.008**
High femoral torsion (> 35°), *n* (%)	16 (70)	47–87	57 (24)	19–31	**<** **0.001**
Femoral torsion, °	42 ± 9	39–46	29 ± 9	28–30	**<** **0.001**
Ischiofemoral distance, cm	1.7 ± 0.6	1.4–1.9	2.8 ± 0.7	2.7–2.9	**<** **0.001**

Values are depicted as mean ± SD (95% CI) if not otherwise noted. 95% CI refers to % for categorical data

*IFI* ischiofemoral impingement, *CI* confidence interval, *SD* standard deviation

^*^ Defined as retroversion index > 30%, posterior wall, and ischial spine sign positive

Bold highlights *p* values reaching statistical significance (*p* < 0.05)

**Table 3:** Single factor and multiple logistic regression analysis with ORs for the probability of IFI in hip MR arthrography

Parameter	Single-factor logistic regression		Stepwise multiple logistic regression	
	OR (95% CI)	*p* value	OR (95% CI)	*p* value
Female sex	27.3 (3.6–205.8)	**<** **0.001**	12.5 (1.6–98.2)	**0.017**
Hip dysplasia LCE < 25°	0.7 (0.3–2.1)	584		
Acetabular overcoverage LCE > 35°	0.9 (0.3–2.7)	0.823		
Acetabular retroversion*	0.7 (0.1–5.2)	0.695		
Cam deformity Alpha angle > 60°	0.3 (0.1–0.8)	**0.012**		
Ischial angle > 127°	13.0 (3.0–56.8)	**<** **0.001**	5.9 (1.3–27.1)	**0.023**
Valgus hip Neck shaft angle > 135°	3.6 (1.4–9.7)	**0.009**		
High femoral torsion > 35°	7.1 (2.8–18.0)	**<** **0.001**	3.9 (1.4–10.4)	**0.008**

Values are depicted as mean ± SD, if not otherwise noted

*LCE* lateral-center-edge angle

^*^ Defined as retroversion index > 30%, posterior wall, and ischial spine sign positive

Bold highlights *p* values reaching statistical significance (*p* < 0.05)

### Comparison of intra-articular lesions in patients with and without IFI

Cartilage damage was significantly less prevalent in the IFI group, occurring in 26% (95% CI: 10–48%) of cases compared to 52% (95% CI: 45–58%) in the control group (*p* = 0.027, Table 4). Regarding labral pathology, the prevalence of labrum tears was similar between the groups, with 87% (95% CI: 66–97%) in the IFI group and 89% (95% CI: 84–93%) in the control group (*p* = 0.732, Table 4 and Fig. 3.

**Table 4:** Comparison of imaging findings between the study groups

Parameter	IFI group (23 hips)		Control group (233 hips)		*p* value
	*n* (%)	95% CI	*n* (%)	95% CI	
Labrum tear, *n* (%)	20 (87)	66–97	207 (89)	84–93	0.732
Base tear	14 (61)	39–80	175 (75)	69–81	0.143
Intrasubstance-/complex tear	6 (26)	10–48	32 (14)	10–19	0.125
Extensive labrum tear	8 (35)	16–57	51 (22)	17–28	0.193
Cartilage damage, *n* (%)	6 (26)	10–48	120 (52)	45–58	**0.027**
Extensive cartilage damage	2 (9)	1–28	60 (26)	20–32	0.077
Ligamentum teres lesion, *n* (%)	7 (30)	13–53	72 (31)	25–37	1.00

Bold highlights *p* values reaching statistical significance (*p* < 0.05)

### Interrater reliability

Interrater reliability analysis for the detection of intra-articular joint degeneration on MR arthrography showed Cohen’s kappa values ranging from 0.55 (95% CI, 0.38–0.73) for the evaluation of the presence and type of labral tear, to 0.84 (95% CI, 0.71–0.96) for the detection of extensive cartilage damage (Supplementary Table 2).

## Discussion

While the prevalence of IFI on MRI has previously been studied in patients aged 50 years or older with hip and/ or buttock pain, with reports ranging from 6% to 18% [19, 20], its prevalence in younger patients remains unclear to date. Our study addressed this gap, reporting a 9% prevalence of IFI in a cohort of young patients (mean age 34 years) evaluated for joint-preserving hip surgery. This finding underscores the growing recognition of IFI as an important cause of hip pain, particularly in young adult females [4, 5, 21].

One of the most important findings of our study was the strong association between IFI and specific osseous hip deformities. Increased femoral torsion, valgus deformity, and increased ischial angle were significantly more prevalent in the IFI group compared to controls. Gómez-Hoyos and colleagues found higher femoral neck version angles in patients with IFI vs asymptomatic controls (21.7° vs 14.1°) [22]. We used the method described by Murphy et al for the measurement method of femoral torsion which yields higher femoral torsion angles [12] and found high femoral torsion in 70% of IFI patients, vs only 24% in controls (*p* < 0.001). More specifically, excessively high femoral torsion > 35° emerged as an independent risk factor of IFI (OR 3.9, *p* = 0.008). This may be explained by a more posterior positioning of the lesser trochanter, potentially narrowing the ischiofemoral space during hip extension and external rotation [23, 24]. Including the distal femoral condyles in the routine MR imaging protocol for measuring femoral torsion is important when evaluating patients for IFI since young patients may benefit from derotational femoral osteotomy for correction of excessively high femoral torsion [21, 25]. The higher prevalence of valgus deformities in the IFI group of 30% compared to 11% in controls (*p* = 0.014) suggests that an increased neck-shaft angle may further contribute to the narrowing of the ischiofemoral space.

The increased ischial angle observed in IFI patients (132° ± 4° vs 126° ± 9°, *p* < 0.001) in our study is consistent with known sex-related differences in pelvic morphology [2, 6]. Bredella and colleagues reported less pronounced but significant differences in their cohort of predominantly middle-aged women (130.6 ± 4.9° vs 128.0 ± 6.2°) compared to controls [2].

Applying a threshold of > 127° for the ischial angle was an independent predictor of IFI, with an OR 5.9 (*p* = 0.023). Similar to other studies [2, 6, 26], female sex also emerged as a strong independent predictor of IFI, with an OR of 12.5 (*p* = 0.017). This sex difference reflects the anatomical adaptations of the wider female pelvis, which reduces the ischiofemoral space and predisposes it to posterior extra-articular impingement [2, 27].

Interestingly, our study found a comparably high prevalence of labral lesions in both the IFI (87%) and control groups (89%, *p* = 0.732), the latter including patients with classic FAI deformities and hip dysplasia. This finding is particularly intriguing as it suggests that IFI can coexist with intra-articular pathology commonly associated with other hip disorders and challenges the notion that IFI is solely an extra-articular problem. It suggests that patients with IFI may be at similar risk for intra-articular pathology as those with more widely recognized hip disorders.

A proposed pathomechanism for this association is the leverage of the femoral head, resulting in overload to the anterior labrum secondary to the ischiofemoral abutment [28, 29]. This is supported by recent observations using MRI in the flexion abduction external rotation position, which demonstrated leverage of the central femoral head and the fovea capitis towards the anterior acetabular rim during provocative maneuvers [24]. This dynamic relationship between extra-articular impingement and intra-articular mechanics may explain the high prevalence of labral lesions in IFI patients, suggesting a more complex interplay between extra- and intra-articular hip pathologies. By contrast, the prevalence of cartilage lesions was much lower in the IFI group compared to the controls (26% vs 52%, *p* = 0.027). Despite the comparable frequency of acetabular dysplasia and pincer deformities, there was a lower prevalence of cam deformities (35% vs 63%, *p* = 0.013) in the IFI group compared to controls. This may be related to the fact that the IFI group was predominantly female (89%). It is well known that cam deformities represent a major risk factor for cartilage damage in FAI [30]. The combination of less severe cam deformities and higher femoral torsion supposedly increases the impingement-free range of hip internal rotation in the IFI group and thus may explain the lower prevalence of cartilage lesions.

Our findings underscore the importance of a comprehensive MRI protocol when evaluating patients for potential IFI [5, 31]. Crucially, this protocol should include fluid-sensitive sequences with fat suppression, which are essential for detecting quadratus femoris muscle edema, a key indicator of IFI. Without these fluid-sensitive sequences, muscle edema might be overlooked, potentially leading to missed diagnoses of IFI. Traction MRA using saline has become the routine protocol in our institution. While saline-based traction MRA using PD-weighted sequences reportedly provides high diagnostic accuracy for detecting chondrolabral lesions [8], this is yet to be shown for standard MRA without traction for which acquisition of T1-w sequences may still be beneficial regarding diagnostic accuracy.

Limitations of our study include its retrospective nature and the potential for selection bias. However, it was our intention to include patients being evaluated for joint-preserving surgery and not include the previously described older patient cohorts [2, 19, 26]. Additionally, our study was limited to static MRI findings as the primary indicator of IFI. This approach may not capture all cases, particularly those without evident muscle edema or those that manifest primarily during dynamic activities. Recent literature has emphasized the dynamic nature of IFI using kinematic MRI and hip MRI in provocative maneuvers to account for the narrowing of the ischiofemoral space most pronounced during hip external rotation [24, 32]. This dynamic aspect of IFI underscores the importance of correlating imaging findings with clinical symptoms and provocative tests, such as the long-stride walking test [33].

In conclusion, our study demonstrates a notable prevalence of IFI in young patients evaluated for joint-preserving hip surgery and identifies specific associated osseous deformities. The high prevalence of labral lesions in IFI patients, comparable to those with FAI and dysplasia, highlights the complex interplay between extra- and intra-articular hip pathologies. These findings emphasize the need for clinicians to consider IFI in the diagnostic workup of young patients with hip pain, particularly in females and those with increased femoral torsion, coxa valga, or increased ischial angle. Future prospective studies incorporating dynamic imaging techniques are warranted to further elucidate the relationship between IFI, osseous deformities, and intra-articular pathology.

## Supplementary information

ELECTRONIC SUPPLEMENTARY MATERIAL

## Data Availability

The datasets generated and analyzed during the current study are available from the corresponding author upon reasonable request.

## Abbreviations

AP: Anteroposterior
CI: Confidence interval
FAI: Femoroacetabular impingement
IFI: Ischiofemoral impingement
LCE: Lateral center edge angle
MRA: Magnetic resonance arthrography
OR: Odds ratio
STIR: Short-tau inversion recovery
