# Supplementary material for: Ischiofemoral impingement in joint preserving hip surgery: prevalence and imaging predictors
Source: Insights Imaging. 2025 Apr 4;16:78. doi: 10.1186/s13244-025-01946-2 (PMC11971088; doi:10.1186/s13244-025-01946-2)
Supplement: Supplementary file 1 — ELECTRONIC SUPPLEMENTARY MATERIAL [file 13244_2025_1946_MOESM1_ESM.pdf]

Ischiofemoral Impingement in Joint Preserving Hip Surgery: Prevalence and Imaging Predictors

ELECTRONIC SUPPLEMENTARY MATERIAL

Supplementary Table 1. Imaging protocol for standard MR arthrography of the hip

Sequence	Repetition Time (ms)	Echo Time (ms)	Inversion Time (ms)	Matrix	FOV (mm)	Flip Angle	Slice Thickness (mm)	Bandwidth (Hz/Px)	Image orientation
PD-w turbo spin echo	2460	13	-	512 x 512	180	150	3	130	coronal/ sagittal/ axial-oblique/ radial
STIR	4360	42	150	384 x 384	360	150	5	161	axial pelvis
T1-w VIBE Dixon	6.7	2.4/ 4.8	-	320 x 320	380	10	3	470	axial pelvis and knee

Proton density weighted (PD-w) images were acquired without fat-saturation.
FOV = field of view. STIR = short-tau inversion recovery. VIBE = volume interpolated breath-hold-examination.

Supplementary table 2. Interobserver reliability for assessment of intraarticular joint degeneration on MR arthrography using Cohen's kappa

**Parameter**	**MR arthrography** (100 hips)
Labral tear (presence and type)	0.55 (0.38 to 0.73)
Extensive labral damage (> 2 hours)	0.80 (0.66 to 0.93)
Cartilage damage	0.71 (0.57 to 0.86)
Extensive cartilage damage (> 2 hours)	0.84 (0.71 to 0.96)
Ligamentum teres lesion	0.62 (0.45 to 0.79)

Values are depicted as quadratic weighted Kappa values (95% Confidence interval).
